# Ovotestis Isolation and Cryopreservation of *Nesiohelix samarangae* (Oriental Snail) as a Snail Model for Conserving Other Endangered Snail Species

**DOI:** 10.3390/biology13040205

**Published:** 2024-03-22

**Authors:** Jukyeong Jeong, Seungki Lee, Jung Kyu Choi

**Affiliations:** 1 Department of Biotechnology, College of Life and Applied Sciences, Yeungnam University, Gyeongsan 38541, Republic of Korea; jjk1016jjk@naver.com; 2 Biological and Genetic Resources Assessment Division, National Institute of Biological Resources, Incheon 22689, Republic of Korea

**Keywords:** ovotestes, *Nesiohelix samarangae*, vitrification, alginate, conservation genetics

## Abstract

**Simple Summary:**

This study aimed to develop a cryopreservation system for *Nesiohelix samarangae* (oriental snail)’s reproductive organ to aid in species conservation. This snail possesses simultaneous hermaphroditism, meaning it has both male and female germ cells in its reproductive organs. Its reproductive glands are divided into acini, containing various stages of sperm development. Ovotestis cryopreservation using 2% alginate encapsulation showed superior viability compared to non-encapsulated methods. This system contributes to the genetic conservation and maintenance of genetic diversity within the species.

**Abstract:**

This study aimed to develop a cryopreservation system for the reproductive organs of *Nesiohelix samarangae* (oriental snail) to support the conservation of their species. The reproductive glands of *N. samarangae* are divided into numerous acini by acinar boundaries. Within each acinus, the presence of spermatogonia, spermatocytes, spermatids, and sperm were observed, indicating various stages of sperm development. The spermatocytes were irregular in shape and possessed large nuclei. Spermatids, on the other hand, were predominantly located within the lumen of the tissue and exhibited densely packed nuclei. Furthermore, sperm with tails attached were observed within the tissue. In order to preserve the oriental snail species, we utilized the vitrification method to freeze the reproductive organs. Comparing the two methods, it was observed that cryopreservation of ovotestis using 2% alginate encapsulation exhibited superior viability following thawing, surpassing the viability achieved with the non-encapsulated approach. In this study, the establishment of a cryopreservation system for the reproductive organs of the oriental snail not only contributes to the genetic conservation of the endangered snail species but also plays a role in maintaining genetic resources and diversity.

## 1. Introduction

*Nesiohelix samarangae* (the oriental snail) is a terrestrial snail belonging to the phylum Mollusca, class Gastropoda, order Pulmonata, superfamily Stylommatophora, and family Bradybaenidae [1]. The reproductive organs of *N. samarangae* are simultaneous hermaphrodites or ovotestes, where both male germ cells, such as spermatogenic cells, and female germ cells, such as ova, coexist simultaneously [2]. Snails consume leaves and small plants, and as they move through the soil, they excrete waste, which enriches the soil with nutrients [3]. In addition, they also eat soil and excrete it, which can also contribute to soil health. The mucus secreted by snails during their movement helps make the soil more fertile [4]. Due to their ability to consume dead insects and plant debris, snails serve as decomposers in the ecosystem, contributing to the ecological nutrient cycling of organic matter [5]. They also play an intermediate role in the food chain as prey for animals such as frogs, hedgehogs, larvae, and certain beetles. Therefore, they can be regarded as a valuable biological resource, contributing to the ecological food web and the overall balance of the ecosystem [6,7].

However, due to urban development and deforestation leading to ecological destruction, soil pollution, and the introduction of invasive species, the population of oriental snails has significantly declined, pushing them to the list of endangered species of concern [8,9,10]. Research on using cryopreservation of reproductive organs for species conservation has been actively conducted in endangered amphibians [11], fishes [12,13], and mammals [14], while studies on snails have been relatively scarce. Research in snail reproductive biology has been conducted on the maturation of oocytes in the ovotestis of the African giant snail (*Achatina fulica*) [15,16] and the microstructure of the ovotestis of *Macrochlamys indica* [2].

In this study, we first successfully isolated the ovotestes and germ cells of oriental snails and observed the developmental stage of the germ cells in the ovotestes through histology. In addition, in order to preserve species for oriental snails, viability was confirmed after freezing the ovotestes using vitrification. Therefore, this research not only provides fundamental data for studying the reproduction of the oriental snail, but also contributes to an innovative tool for the genetic conservation of other endangered snail species.

## 2. Materials and Methods

### 2.1. Breeding Environment of Nesiohelix samarangae

Oriental snails (*Nesiohelix samarangae*) were reared in an environment of 14 h light:10 h dark. The initial size was 0.5 to 1 cm, and specimens were reared in 34 cm × 20.5 cm × 25 cm plastic boxes. Cocopeat, a snail soil, was laid to a depth of about 3 cm on the floor, and moist moss was provided to maintain a humid environment. The temperature was maintained at 24 to 28 °C. 

### 2.2. Reproductive Organ Isolation of Nesiohelix samarangae

In order to alleviate pain in snails prior to euthanasia, a two-step procedure was followed. Initially, anesthesia was induced using 5% ethanol, followed by euthanasia using 70% ethanol. Scissors were used to carefully cut off the shells of *N. samarangae* from the outside. After all the shells were removed, the film was peeled off using a scissor and forceps and the gonads located inside were recovered. After the gonads were recovered, the gonad tissue was transferred to 35 mm Petri dishes containing 2 mL of DPBS (Wellgen, Gyeongsan, South Korea).

### 2.3. Histology of Reproductive Organ of Lissachatina fulica and Nesiohelix samarangae

The reproductive organs were fixed with 4% paraformaldehyde, embedded into paraffin blocks, and then sectioned at 5 μm using a microtome. The hematoxylin and eosin (H&E) staining were carried out for histological analysis. Finally, they were observed under a microscope.

### 2.4. Vitrification of Reproductive Organs from Nesiohelix samarangae

Alginate solution was prepared by adding 0.02 g of lyophilized alginate to 1 mL of deionized water, along with 0.25 M mannitol and 10 mM HEPES. Reproductive organs were washed in DPBS, immersed in the alginate solution for about 30 s, transferred to the CaCl_2_ solution, and allowed to stand for approximately 1 min for encapsulation. We loaded cryoprotective agents (CPAs) into them in two steps. This was performed by first incubating encapsulated reproductive organs (RO) with a pre-equilibrium solution made of 10% dimethyl sulfoxide (DMSO), and 10% ethylene glycol (EG) for 10 min. Next, the organs were transferred into 500 μL vitrification solution made of 20% DMSO and 20% EG for 2 min and put on a copper grid (Ted Pella, Redding, CA, USA). The RO-laden copper grid was then plunged into liquid nitrogen and held there for at least 1 min and thawed sequentially for 3 min in 0.5 M, 0.25 M, 0.125 M, and 0 M sucrose. After thawing, the cells were isolated from chopped the tissues with 1 mL syringe, and measured cell viability using live/dead and nuclear staining with fluorescein diacetate (FDA, live cells in green), propidium iodide (PI; dead cells in red) (2 μg/1 mL) and hoechst 33258 (nuclear in blue) (1 μg/1 mL), respectively. The same procedure was carried out for the control group without RO.

## 3. Results

**Figure 1:**
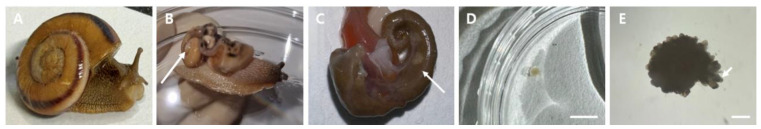
Reproductive organs isolation from *Nesiohelix samarangae*. (**A**) *N. samarangae*. (**B**) The appearance of a snail without its shell. (**C**) The location of the ovotestis (white arrow). (**D**) The morphology of the ovotestis, colored whitish. (**E**) The ovotestis with oval-shaped acini (white arrow). Scale bar = 5 mm (**E**) The morphology of the ovotestis with acini. Scale bar = 0.5 mm.

We isolated the ovotestis, which is a unique reproductive organ, from *N. samarangae*. The shell of this species is colored a yellowish-brown hue, with two distinct dark brown stripes running along its surface (Figure 1A). This creates a visually striking appearance that is characteristic of this particular species. The ovotestis is located on the inner side (opposite side) of the apex (white arrow) (Figure 1B). This organ, which can also have a whitish coloring, is situated at the apex (top) of the shell and is internalized within the digestive gland (Figure 1C). The ovotestis was isolated for cryopreservation, and it was revealed to consist of clusters of oval-shaped acini, which can be identified by the white arrow in Figure 1D,E.

**Figure 2:**
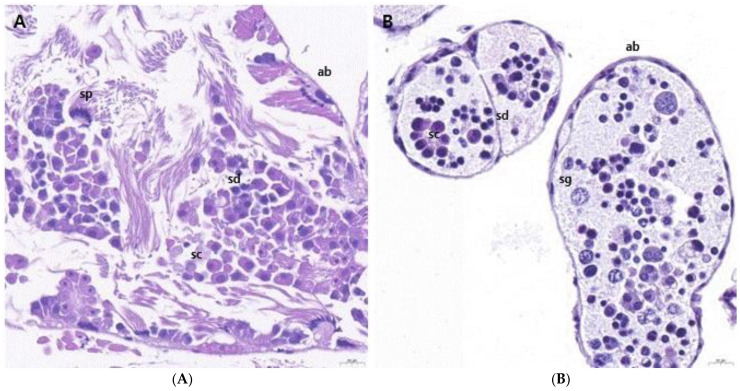
Histology of ovotestes of *Lissachatina fulica* and *Nesiohelix samarangae*. (**A**) The ovotestes of *L. fulica* and *N. samarangae* are composed of numerous oval-shaped acini, within which spermatogonia, spermatocysts, spermatids, and sperm were observed. (**B**) Spermatocytes with large nuclei were found toward the periphery of the acini and densely packed spermatids with concentrated nuclei. ab = acinar boundary; sg = spermatogonia; sc = spermatocyte; sd = spermatid; sp = sperm. Scale bars = 20 μm.

We performed H&E staining to examine the presence of germ cells within the ovotestes of *L. fulica* and *N. samarangae*. The ovotestis of *L. fulica* was found to be composed of numerous oval-shaped acini. The acini were demarcated by acinar boundaries, and it was possible to identify the presence of spermatocytes and spermatids within the interior. The spermatids were found to be located towards the lumen, compared to spermatocytes, and their nuclei appeared more densely packed within the acini (Figure 2A). In addition, it was possible to observe sperm with tails, indicating the presence of mature spermatozoa following spermatogenesis. In *N. samarangae*, spermatocytes with larger nuclei were found towards the periphery of the acini. Within the acini, spermatids with densely packed nuclei were observed. However, unlike in *L. fulica*, no sperm with tails, indicative of post-spermatogenesis, were observed within the interior (Figure 2B).

**Figure 3:**
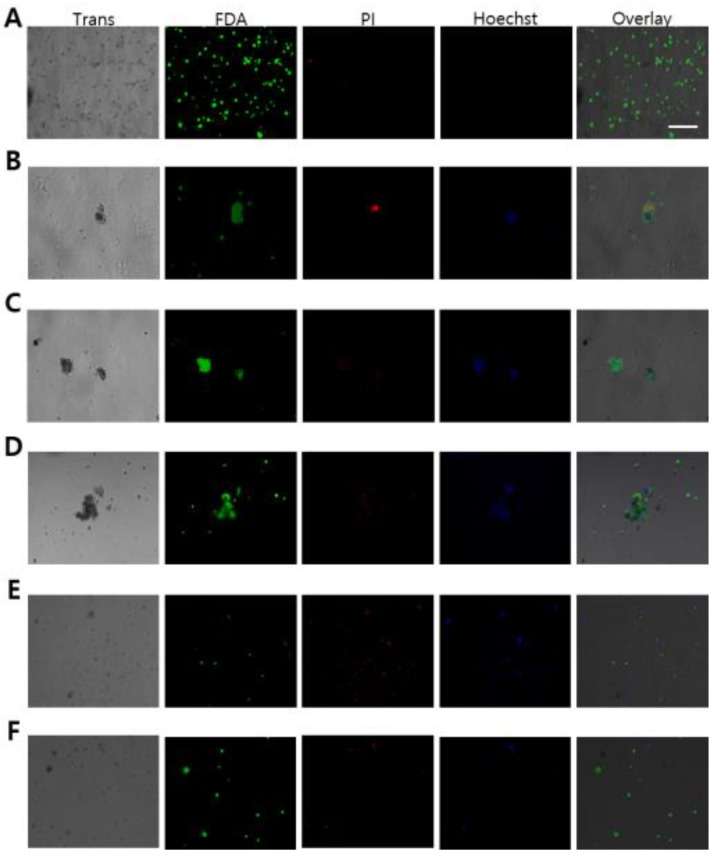
Cell viability of ovotestes in *Lissachatina fulica* and *Nesiohelix samarangae* after vitrification. To validate cell viability in the ovotestis, assessments were conducted using FDA (live cells in green), PI (dead cells in red), and Hoechst 33258 (nuclear in blue) staining. (**A**–**C**) *Lissachatina fulica* (**D**–**F**) *Nesiohelix samarangae* (**A**,**D**) negative control (no freezing). (**B**,**E**) Vitrification. (**C**,**F**) Alginate-encapsulated ovotestis vitrification. Scale bars = 100 μm.

In order to compare the effects of cryopreservation between alginate encapsulated ovotestis (AEO) and without AEO in *L. fulica* and *N. samarangae*, the viability of germ cells in the ovotestis was confirmed using live/dead staining after thawing. Compared to the group without AEO, the AEO group exhibited a greater presence of live germ cells that exhibited fluorescenin diacetate (FDA) staining (live cells in green), along with a reduced level of propidium iodide (PI) staining (dead cells in red), indicating decreased cell mortality (Figure 3). The diminished PI staining suggests a decrease in cell death within AEO, thereby implying a potential protective effect of alginate encapsulation against cryopreservation induced cellular damage.

## 4. Discussion

We utilized *N. samarangae* (oriental snails), listed as a species of concern, as an alternative model for the conservation of endangered snails, due to the difficulty in obtaining endangered species of snails. Furthermore, due to the scarcity of research on the reproductive organs and reproductive cells of *N. samarangae*, comparative studies were conducted with *L. fulica*. We have established methods for isolating the reproductive organs and sperm cells of *N. samarangae*. By performing hematoxylin and eosin (H&E) staining on the ovotestis, we were able to observe the presence of sperm cells within the ovotestis and their developmental stage. In the case of snails, which are hermaphrodites, both sperm and eggs are expected to be present in the ovotestis. However, in the ovotestis of both *L. fulica* and *N. samarangae*, the presence of eggs was not observed. In younger individuals, the predominant process may be the formation of sperm [2]. Although we detected the presence of spermatids in the ovotestis of *N. samarangae*, we were unable to observe the final stage of sperm. This suggests that the process of sperm maturation and development may not have been completed at the time of our observations. Further investigation is needed to understand the exact mechanisms and timing of germ cells maturation in the snail.

Firstly, we achieved successful preservation of the reproductive organs of *N. samarangae* through the application of vitrification. In addition to significantly elevated survival rates of reproductive cells when utilizing the vitrification method in conjunction with alginate encapsulation, the prevention of ice crystal formation within cells before and after freezing, provided by alginate encapsulation helps protect the internal structure and cell membranes from damage, further enhancing cell survival rates [17,18,19]. We have successfully developed a method for isolating and cryopreserving ovotestes from *N. samarangae*, and this technology could potentially be applied to the conservation of other endangered snail species. Preserving endangered snails holds significant ecological importance, as they maintain the balance of nature by performing unique roles in the ecosystem. In addition, conserving endangered species is imperative to sustain biodiversity and protect valuable genetic resources that can be used to benefit humanity [20,21].

However, despite the successful cryopreservation of the ovotestes of the snail, the use of high cryoprotectant (CPA) concentrations during vitrification can lead to toxicity in reproductive cells. In the future, we plan to conduct genetic analysis and in vitro studies on the developmental processes of reproductive cells to assess the toxicity of CPAs and their impact on reproductive cells before and after thawing. Therefore, this research not only provides crucial data for studying the reproductive cells and organs of snails, but also contributes to the conservation of endangered snail species.

## 5. Conclusions

We have successfully developed techniques to isolate the reproductive organs and germ cells of snails, which can contribute to breeding programs and the long-term survival of the species. The findings highlight the potential for using cryopreservation techniques to conserve reproductive capacity and genetic diversity in endangered species. Therefore, this research contributes to the broader field of conservation biology and serves as a foundation for further studies in the preservation of the oriental snail and other endangered species.

## Data Availability

The authors are willing to provide the data upon reasonable request.
